# Structural Health Monitoring in Composite Structures: A Comprehensive Review

**DOI:** 10.3390/s22010153

**Published:** 2021-12-27

**Authors:** Sahar Hassani, Mohsen Mousavi, Amir H. Gandomi

**Affiliations:** 1 Department of Civil Engineering, Faculty of Engineering, Ferdowsi University of Mashhad, Mashhad 9177948974, Iran; sahar.haassani@gmail.com; 2 Faculty of Engineering and IT, University of Technology Sydney, Ultimo 2007, Australia

**Keywords:** composite structures, fracture mechanisms, structural health monitoring, smart composite, advanced technology systems

## Abstract

This study presents a comprehensive review of the history of research and development of different damage-detection methods in the realm of composite structures. Different fields of engineering, such as mechanical, architectural, civil, and aerospace engineering, benefit excellent mechanical properties of composite materials. Due to their heterogeneous nature, composite materials can suffer from several complex nonlinear damage modes, including impact damage, delamination, matrix crack, fiber breakage, and voids. Therefore, early damage detection of composite structures can help avoid catastrophic events and tragic consequences, such as airplane crashes, further demanding the development of robust structural health monitoring (SHM) algorithms. This study first reviews different non-destructive damage testing techniques, then investigates vibration-based damage-detection methods along with their respective pros and cons, and concludes with a thorough discussion of a nonlinear hybrid method termed the Vibro-Acoustic Modulation technique. Advanced signal processing, machine learning, and deep learning have been widely employed for solving damage-detection problems of composite structures. Therefore, all of these methods have been fully studied. Considering the wide use of a new generation of smart composites in different applications, a section is dedicated to these materials. At the end of this paper, some final remarks and suggestions for future work are presented.

## 1. Introduction

Structural health monitoring (SHM) seeks to perform several tasks, such as damage detection, localisation, and quantification, to maintain the integrity of an entire structure. Comparatively, baseline-dependent SHM techniques need data from both “healthy” and “damaged” states of structure, whereas baseline-independent SHM techniques seek to identify damage through studying structural response to some natural or synthesised forces. Identifying damage early is desirable so that suitable maintenance procedures can be undertaken, whereby the structural integrity and reliability can be ensured. SHM systems comprise the three following main elements:

- A sensing technology that can be deployed on a structure permanently is used so that structural response data can be recorded and transmitted to a control center to monitor the health condition of the structure. However, traditional non-destructive damage testing is more reliant on scheduled monitoring of the structure at a certain time and location.
- The recorded data are required to be processed through high-performance computing facilities in the control center for real-time condition monitoring of the structure. This was made possible by the advent of high-performance PCs in the mid-1980s.
- Robust algorithms needed to study recorded vibration data for damage must be resilient to several factors, such as measurement noise and Environmental and Operational Variations (EOV) effects. Advanced machine learning, deep learning, and signal processing algorithms have made the development of such methods possible.

The need for resilient materials has been increasing more than ever due to advancements in different fields of engineering over the past century. As such, composite materials have emerged and have been used in many applications. The idea of composite materials was initiated based on mimicking natural materials such as wood. They have been widely used ever since their emergence in different fields of engineering, including civil infrastructures as well as the automotive and aerospace industries. This is mainly due to several outstanding and excellent properties of such materials, including increased stiffness, strength, corrosion resistance, fatigue life, and wear resistance along with enhanced thermal properties and reduced weight. Composite materials are usually obtained from combining two or more components to achieve the aforementioned enhanced engineering properties.

Existing damage in a composite can adversely affect its performance and, if not identified and fixed in time, can lead to catastrophic consequences, such as total destruction of the structure. There are a variety of failure mechanisms in composite structures, which usually develop either during the manufacturing process, such as design errors and overheating, or while in service, such as static overload, shock, and fatigue [1,2,3]. These mechanisms include fiber failure, buckling, matrix cracking, and delamination. Fiber failure is known to be the simplest failure mechanism in composite structuresto detect and usually appears when the excitation loads applied to the composite structure cause fractures in the fibers. Matrix damage, on the other hand, usually appears in several forms, including voids, cracks between fibers within lamina, or even as a single composite layer that is an intralaminar form of defect [4,5]. Another possible form of failure is buckling, which commonly appears as shear or compression [6,7]. A main failure mechanism is delamination, known to be one of the greatest “weaknesses” of laminated composites [1,8]. Delamination can spreed through a composite laminate, resulting in catastrophic consequences if not discovered and fixed swiftly. The stiffness of composite structures can be vastly compromised by damage, where in some cases, it might result in total destruction of the structure. Therefore, it is important to monitor these structures for damage while lowering the maintenance costs. This prompts further the development of structural damage-detection systems to obtain efficient and reliable damage-detection methods. One strategy is to develop advanced Non-Destructive Testing (NDT) technologies that can detect such local abnormalities in composite structures. There are different types of NDT techniques used for the structural damage identification of composite structures, some of which include visual testing (VT) or visual inspection (VI), ultrasonic testing, thermographic testing, infrared thermography testing, radiographic testing, acoustic emission testing (AE), acousto-ultrasonic, shearography testing, optical testing, liquid penetrant testing, magnetic particle testing, and electromagnetic testing.

Advancements in SHM techniques for composite structures widely favor the methods developed for other structures. Some examples of such methods can be found in [9,10,11,12,13]. Some of these methods are also listed in Table 1.

This study presents a comprehensive review of some key aspects of damage detection in composite structures, including

1. Laminated composite structures;
2. Types of failure modes in such structures;
3. Various damage-detection techniques that are suitable for such structures as well as their key properties; and
4. Advantages and disadvantages of such techniques. At the end of this study, some updated guidelines for undertaking smart monitoring systems for composite laminate structure are outlined.

## 2. Composite Structures

Common types of engineering materials include metals, polymers, ceramics, and composites. Among these, composite materials are often a better alternative for traditional materials, such as metals, ceramics, and polymers due to their light weight, corrosion resistance, high strength and stiffness, ability to withstand high temperatures, and simple manufacturing process [23,24]. Composite structures are used in a range of different industries from aerospace, marine, aviation, transport, and sports/leisure to civil engineering. For example, advanced composite materials have been used in different structures regarding the above industries, such as rotor blades, aircraft main body, and wing skins.

Laminated composites usually consists of a couple of ply termed as lamina. Each lamina generally consists of two substances: (1) the matrix, and (2) the reinforcement material or fiber, which is immersed in the matrix. Generally, composite materials are made of a base material (matrix) and a reinforcement material (fiber) [24,25,26]. Fiber-reinforced composite (FRC) materials are composed of high-strength fibers that are embedded in a matrix for two main reasons: (1) to hold the fibers in place and (2) to prevent the fibers from exposure to destructive environmental conditions, such as humidity. The different types of composite textures pertain to fibrous composites, laminated composites, particulate composites, symmetric laminates, and unsymmetrical laminates.

**Figure 1:**
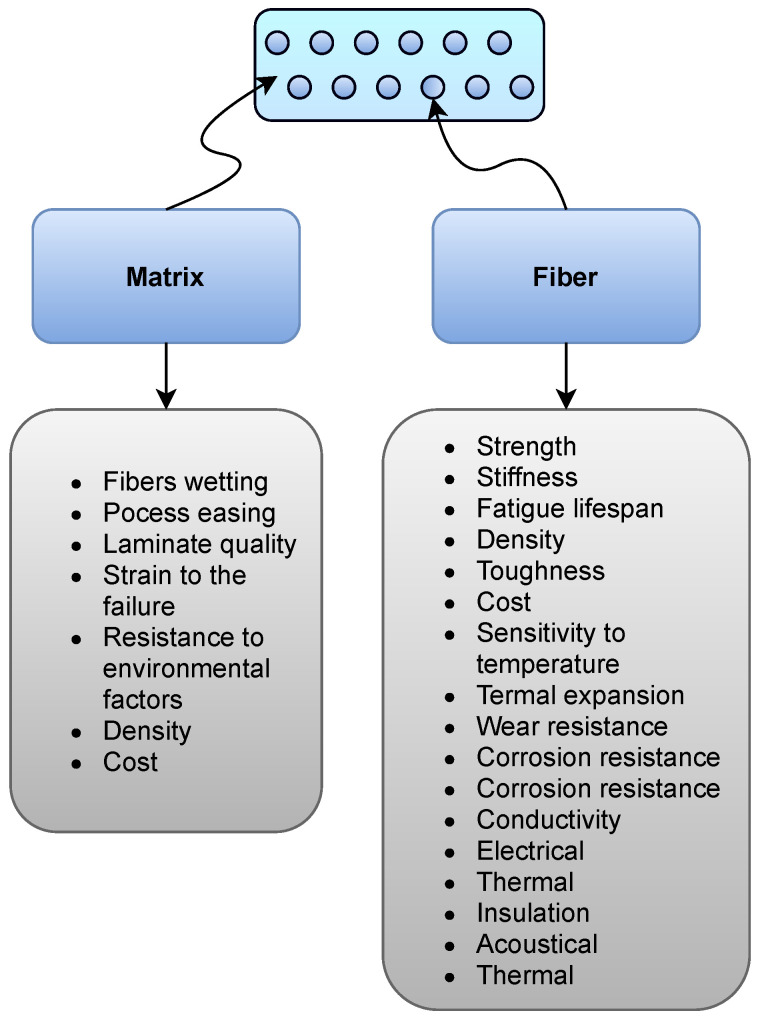
The contributions of matrix and fibers to different properties of a ply.

Figure 1 shows the contributions of the matrix and fiber to different properties of a ply in composite laminates.

- Fibrous Composites:
  Fibrous composite is a type of composite materials that includes fibers integrated with a matrix, owing its remarkable stiffness and strength to the fibers. Fibers can be classified based on their length into long and short fibers. While long fibers are usually produced in straight form or woven form, short fibers, also known as whiskers, possess better strength and stiffness properties. The geometrical properties of a fiber are usually characterised by a high length-to-diameter ratio as well as its near crystal-sized diameter. The effectiveness of a fiber is, however, determined by its strength-to-density and stiffness-to-density ratios. Fibers can effectively improve the fracture resistance of the matrix [27], and the long-dimension reinforcement made by fibers stalls the growth of the cracks initiating normal to the direction of reinforcement.
- Laminated Composites:
  Laminated composites consist of several layers of different materials (at least two) bonded together. Since layers are usually very thin individually, they are combined through lamination to achieve a material with better mechanical properties. Various orientations of the layers are typically used to form a multiply laminated composite suitable for engineering applications. Some examples of laminated composites include bimetals, clad metals, laminated glass, plastic-based laminates, and fibrous composite laminates [28].
  A hybrid class of composites, called laminated fiber-reinforced composites, involves both fibrous composites and lamination techniques. The fiber direction of each layer of fiber-reinforced composites is typically oriented in a direction different from the direction of other layers in order to achieve strength and stiffness in different directions. Thus, the layering of such composites can be tailored based on specific design requirements [29].
- Particulate Composites:
  Particulate composites, such as concrete, consist of particles of different materials with different shapes, sizes, or configurations that are randomly suspended in a matrix. However, unlike fibers, particulate composites are not usually of long dimensions (with the exception of platelets) but instead are regarded as isotropic materials. Similar to a matrix, particles can be composed of different types of materials, including metallic and nonmetallic. As such, there are four possible combinations of fibers and matrices in terms of the type of material used in each one: (1) metallic particles in nonmetallic matrix, (2) nonmetallic particles in metallic matrix (metal matrix composites), (3) nonmetallic particles in nonmetallic matrix, and (4) metallic particles in metallic fibers. Particulate composites are meant to reduce the cost of integrating composites with fibers [30]. Notwithstanding, they typically do not exhibit the strong load-bearing capability of fibrous composites and are not typically resistant to fracture.
- Symmetric Laminates:
  Symmetric laminates are a laminated composite that is symmetric in geometry and material with respect to the geometrical middle surface. Therefore, the layers that make up a symmetric pair possess the same properties. Symmetric laminates are more common compared with unsymmetrical laminates [31].
- Unsymmetrical Laminates:
  Unsymmetrical laminates are not symmetric with respect to their middle surface. They are used in many applications, depending on the design requirements [32].

Often times, various types of composite textures can be mixed to obtain six different kinds of composite materials as follows:

- Symmetric–fibrous composites;
- Symmetric–laminated composites;
- Symmetric–particulate composites;
- Unsymmetrical–fibrous composites;
- Unsymmetrical–laminated composites; and
- Unsymmetrical–particulate composites.

**Figure 2:**
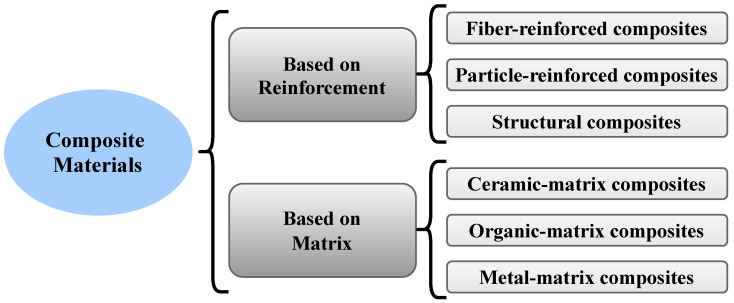
The classification of the composite material.

The load is mainly carried by the fibers that act as reinforcement, while the roles of the matrix are (1) to hold the fibers in place and (2) to transmit the load to the fibers. Typically, fibers are composed of carbon, glass, aramid, boron, and silicon carbide, whereas the matrices are usually made from polymers such as epoxies and polyimides [32]. Figure 2 shows the classification of composite materials based on the type of reinforcement and matrix. Therefore, the properties of a composite are generally determined by the following factors:

1. Fiber properties;
2. Matrix properties;
3. Fiber Volume Fraction (FVF), which is defined as the ratio of fiber to matrix; and
4. Arrangement of fibers in the composite, such as geometry and orientation.

The density, stiffness, and strength of the matrix is lower than those of the fibers. The combination of the matrix and fibers usually offers very high strength and stiffness while maintaining low density [26].

For further details about the classification of composite structures, the readers are referred to [33,34,35,36].

### 2.1. Failure Mechanisms of Composite Structures

**Figure 3:**
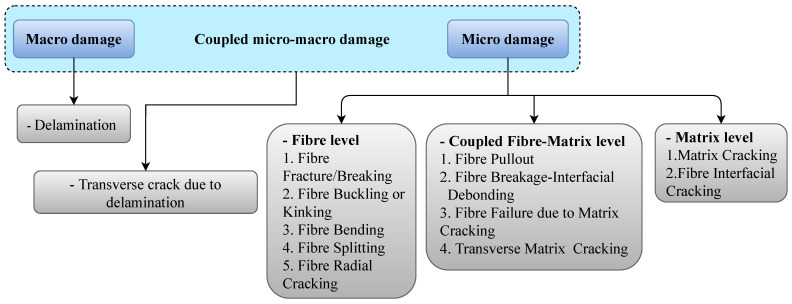
Types of damage in composite structures.

Various types of defects can occur in composite structures, which can be classified based on the size and component of the effected composite structure, as illustrated in Figure 3. Some of the most critical types of damage are those caused by cyclic loading (fatigue damage) or impact loading. Such damage can significantly reduce the residual strength in a part of a composite structure, depending on their type and size [36]. Damage can occur in a composite structure in different forms, ranging from defects in the matrix or fiber to other forms of damage such as a breakage of elements or failure of attachments that are either bonded or bolted to the body of the structure [5]. The extent of damage determines the remaining service life of a composite component and is thus considered a factor to identify the damage tolerance of the component. While some types of damage can have very little effect on the residual strength, they can become more severe over time when combined with other factors, such as environmental and operational effects [37,38].

Impact damage can reduce the compression, shear, and tensile strength of composite materials. As such, the compressive residual strength of the laminated composite material is dependent on the extent of delamination and fiber failure produced by transverse impacts. Fiber failure can subsequently affect the tensile residual strength of the material. However, the effect of impact damage can vary based on the specific design and application of the composite member. For example, in aircraft systems, impact damage can decline the resistance and integrity of composite components to the environmental factors, such as moisture. As such, the core of sandwich panels with thin face sheets may be subjected to moisture after the impact, or the impact can bring about fuel leaks in stiffened wing panels. Therefore, a good understanding of these effects can guarantee a safe and economic application of composite materials.

Table 2 lists some studies that investigate common failure mechanisms in composite structures.

Some more details about failure mechanisms in composite materials can be found in [10,42,43,44].

### 2.2. Environmental Variations Effects

One pertinent factor to be considered when designing a composite component is the environment that the component is exposed to during service time. This is mainly due to the fact that the performance of composite members is significantly affected by environmental factors. There are several environmental factors that can have such effects, with temperature and moisture being the most important for polymer composites. For example, the modulus and strength of the polymer matrix are highly affected by temperature variations, which can further affect the mechanical properties of the lamina and laminate. While the modulus and strength of the matrix can be reduced by elevated temperature, extreme cold conditions can trigger brittle behaviour in some resin systems [45,46,47,48]. However, the extent of this event highly depends on the type of resin and, more generally, all other materials used in the design of the composite component. For example, the effect of temperature on glass or carbon fibers is less than that on some organic fibers, such as aramid. Likewise, increased moisture content can decrease some mechanical properties of materials, such as the resin’s modulus and strength. Moreover, matrix swelling is another effect caused by moisture uptake, resulting in increased residual stresses within the laminate. Except for most spacecrafts, moisture swelling effects are not as severe as those pertaining to temperature and, therefore, are usually neglected at the design stage.

Table 3 outlines the effect of different environmental, operational, and damage mechanisms on the mechanical properties of composite structures based on reviewing References [33,34,49,50,51]. For instance, the composite material stiffness is highly sensitive to the temperature and moisture variations as well as the presence of fiber cracks. Another factor that is highly sensitive to moisture, as an environmental effect, is the mass of composite components. As such, the boundary formation is the item least influenced by the environmental variations, i.e., temperature and humidity. The mechanical load and electromagnetic radiation have relatively moderate effects on composite material conductivity. However, their impact on other mechanical properties of the composite structure is negligible.

Table 4 indicates the review of several studies on the environmental and operational effects on different types of structures. Some further references on this topic include [52,53,54,55].

## 3. SHM of Composite Structures

Structural health monitoring (SHM), as a well-established tool, is currently used extensively for damage diagnosis in different types of composite structures, such as bridges. SHM methods can be categorised into two groups in terms of the extent of the area they are applied to on a structure: local and global techniques. Global techniques are of more interest when it comes to monitor a large area on structures, whereas local methods, also termed non-destructive evaluation (NDE) techniques, have been widely used for damage identification of different structures such as composite materials.

Non-destructive testing (NDT) refers to a family of damage-identification methods that do not pose damage onto the structure under investigation. As such, they are valuable techniques in terms of saving money and time in system evaluation. Alternatively, these techniques may be termed nondestructive examination (NDE), nondestructive inspection (NDI), or nondestructive evaluation (NDE) [69,70,71,72,73]. The advantages, limitations, and range of applications of different NDT methods are listed in Table 5. Accordingly, thermography and ultrasonic testing are the most suitable NDT methods for damage identification in composite materials. NDT aims to detect the presence of and to characterise damage in the interior or on the surface of materials without cutting or piercing through the materials that can otherwise lead to changing the material properties. NDT techniques can be categorised in several ways based on the type of the composite to be tested and testing conditions.

According to Table 5, NDT is widely employed in forensic engineering of different systems, including mechanical engineering, petroleum engineering, electrical engineering, civil engineering, systems engineering, aeronautical engineering, medicine, and art [86,86]. For instance, medical imaging techniques, such as echocardiography, medical ultrasonography, and digital radiography, are NDT techniques that have had a profound impact on medicine.

Ultrasonic testing (UT) techniques belong to another family of NDT techniques that are used to investigate materials by studying the propagation of ultrasonic waves. Typically, UT devices transmit very short ultrasonic impulses with center frequencies ranging from 0.1 to 15 MHz and, in some cases, up to 50 MHz. The recorded signals at the receiver side are studied for internal flaws or in order to characterize materials [5,87,88,89]. For example, UT is used to measure the thickness of the test object to determine the extent of corrosion in a piping system.

Shearography or speckle pattern shearing interferometry is an NDT technique that uses coherent light or coherent sound waves for the quality assessment of materials in different problems, such as nondestructive testing, strain measurement, and vibration analysis. It has a wide range of applications in the aerospace and wind turbine industries, among other areas [5,29,90,91]. The shearography techniques present several advantages over traditional NDT techniques, including (1) being capable of testing large area on the structure (up to 1 m^2^ per minute [92]), (2) providing contactless techniques, (3) being relatively insensitive to environmental variations effects, and (4) performing well on honeycomb materials [93].

Eddy-current testing (ECT) is an electromagnetic NDT method that exploits electromagnetic induction in conductive materials for the detection/characterisation of surface and sub-surface defects [94].

Thermographic inspection is a technique used to monitor the thermal changes in the surface of an object. It can be also used to provide images from thermal patterns on the surface of an object. The infrared thermography technique is non-intrusive and contactless and is used to provide mapping from thermal patterns (thermograms) on an object’s surface through an infrared detector [95].

Radiographic Testing (RT), on the other hand, is an NDT technique to inspect the interior of a material for hidden flaws. In order to penetrate into the material, RT applies short-wavelength electromagnetic radiation [96], which can be produced by some equipment, such as X-ray machines. To provide high-energy photons, the machine is equipped with a source of radioactive material, such as Ir-192; Co-60; or in some rare cases, Cs-137. Neutron imaging is a variant of radiographic testing that produces an image with neutrons, while neutron radiography is a technique that applies neutrons, instead of photons, to penetrate through materials. The neutron attenuation determines the properties of the obtained image. Despite some similarities, it might not be possible to see some details in the resulting images of neutron radiography that could be otherwise detected through X-ray imaging techniques, and vice versa. For instance, neutrons can pass through lead and steel easily but not through plastics, water, and oils [97]. The thickness or composition of a material is determined by measuring the variations in the radiation detected in an opposite side of the material as waves penetrate and pass through.

**Figure 4:**
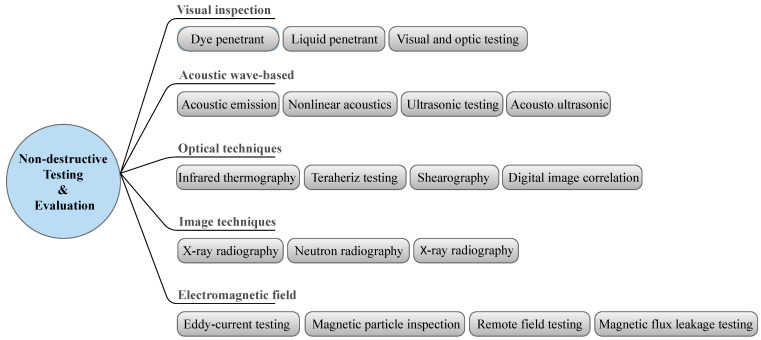
Categories of different non-destructive testing and evaluation techniques (NDTE).

**Figure 5:**
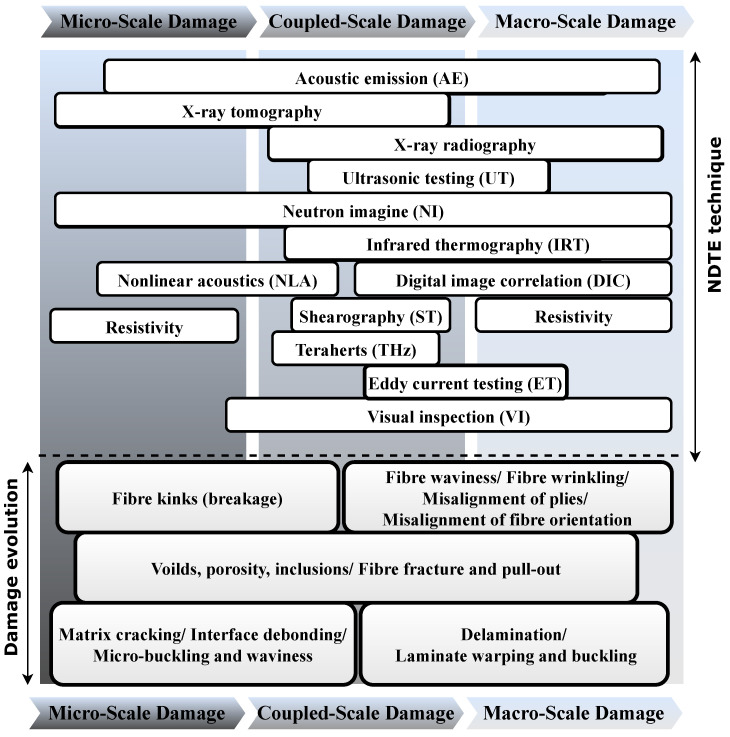
The range of damage to which different types of NDTE techniques can be applied.

Electromagnetic testing (ET) is a family of NDT techniques that monitors the electromagnetic response of a test object by applying electric currents and/or magnetic fields inside the object. Figure 4 lists different types of non-destructive testing and evaluation techniques (NDTE) along with their subcategories. Each of these techniques can be applied to a specific range of damage in composite structures, as shown in Figure 5.

As a main disadvantage of these techniques, the evaluation process cannot be carried out without any prior knowledge about the approximate location of the damage. The SHM system should ideally fulfill the following requirements:

- Cheap;
- Enables continuous assessment;
- Can detect low level damage;
- Can detect different damage types;
- Resilient to ambient loading conditions;
- Resilient to measurement noise; and
- Resilient to environmental variations.

### 3.1. Characteristics of Sensors for SHM

Any SHM system requires a data-collection mechanism, for which different types of sensors can be selected depending on the type of data required for damage detection. Some commonly used sensors include strain gauges [98], accelerometers [99], temperature gauges [100], acoustic emission sensors [101], and fiber optic-based sensor systems [102]. Several factors to be considered prior to select sensors for an SHM system are described as follows:

- Type of sensors;
- Sensor cost(s);
- Number of sensors and their installation procedure;
- Damage protection against mechanical and chemical factors;
- Reduction in the effect of noise;
- Data-collection procedure; and
- Sensitivity of sensors to long-term environmental effects, such as moisture and humidity.

Therefore, sensors need to be protected against harsh environmental effects for obtaining decent measurements. Sometimes, powerless sensors may be desired [103,104,105,106], especially for long-term condition monitoring of structures. These sensors do not require a source of power to operate and are usually equipped with an energy-harvesting mechanism. Some of the main characteristics of sensors are listed in Table 6.

The type of sensor to be employed for damage detection is determined based on the type of data to be measured. Table 7 presents different types of sensors that could be used for monitoring different mechanical properties of a component. Additionally, some criteria to be considered prior to sensor selection are listed in Table 8 based on the authors’ extensive review of the literature.

Optimal sensor placement is an important task that needs to be addressed properly for any successful SHM system. As such, the extraction of sufficient and useful information from the structural response to some applied forces can be guaranteed through the deployment of the sensor network on the identified optimal locations on the structure [127].

### 3.2. Damage Detection Using Ambient Vibration Data

Ambient vibration data provide information on the functions of a structure’s physical properties and, thus, are widely used for damage identification in different types of structures. Damage can reduce the mass and stiffness of a structure while increasing its damping ratio locally. Hence, any information about damage can be retrieved from studying structural modal data. Usually, information about all modal parameters, such as natural frequencies, mode shapes, and modal damping ratio or some combinations of them, are employed for damage detection. Among all structural properties, damping and mass are, respectively, the most and the least sensitive parameters to damage [128,129,130,131,132]. Since damping cannot be easily modelled as with mass and stiffness, proportional damping is a preferred alternative often used for damage detection [133,134,135]. Surface measurements of a vibrating structure can carry information about the health condition of internal members. Hence, the majority of such methods exploit lower-frequency modal data to characterise the global behaviour of structures. Additionally, measurement points can be customized in these techniques due to their global nature. These methods also favour cheap-to-obtain and easy-to-extract properties of the modal information.

However, these methods present some limitations:

1. Sensitivity only to some particular forms of damage;
2. Usually requiring baseline data extracted from a healthy model of the structure to be compared against data obtained from a damaged state for damage characterisation;
3. Succumbing to some structural conditions, such as closely situated eigenvalues, a phenomenon occurred in composite structures [136];
4. Requiring large data storage capacity derived from complex structures, such as composite structures; and
5. Not being capable of extracting information about small defects from global features.

Table 9 summarises different modal features used for damage detection of composite structures along with the type of damage that can be detected and the advantages and disadvantages of each based on the authors’ extensive review of the literature.

#### 3.2.1. Natural Frequency

It is known that damage can reduce the stiffness of a structure, causing its natural frequencies to decline. Therefore, such natural frequencies provide good parameters to be studied for damage detection and classification. Classical vibrational measurement data are usually employed for the identification of structural natural frequencies, thus allowing for the procedure to be a very cheap experimental practice. Therefore, being cheap and easy to measure, natural frequencies are an easy choice for conducting damage detection. Another advantage comes from the level of confidence in the accurate measurement of frequencies, where uncertainties in the measured frequencies can be considerably reduced by a perfect control of the experimental conditions. Moreover, the selection of adequate measurement points for efficient detection of the changes in frequencies can be performed by studying numerical models, such as finite element models, which further enhance the simplicity of identifying the damage location and severity. According to Doebling et al. [137], the first attempt to identify damage by studying the shift in structural natural frequencies was made by Lifshitz and Rotem [138]. Specifically, the latter authors analyzed the shifts in the natural frequencies made by changes in the dynamic moduli for damage detection of elastomers. Notwithstanding, it is known that natural frequencies are highly sensitive to environmental effects, such as temperature fluctuations.

For more information about damage detection in composite structures via natural frequencies, the readers are referred to [139,140,141,142].

#### 3.2.2. Mode Shapes

Mode shapes are relatively less influenced by environmental effects than frequencies, making them a better choice for damage assessment of structures [143]. Moreover, this type of spatial information has been proven to enable damage localisation (Level 2 as per [144]). Modal Assurance Criterion (MAC) is a statistical technique developed on the basis of structural mode shape data and has been widely used for damage detection [145]. This method favors the orthogonality property of eigenvectors. Coordinate Modal Assurance Criterion (COMAC) is an advanced version of MAC that uses modal node displacement for damage detection and localisation [145]. It has been demonstrated that MAC and COMAC can be successfully used to detect and localise different types of damage [146]. COMAC, either alone or in conjunction with other methodologies, seems to be a popular damage detection method across different disciplines of engineering. Table 10 presents some recent developments in the application of mode shapes for damage detection of composite structures.

For more information about damage detection in composite structures via modal shapes refer to [151,152].

#### 3.2.3. Modal Curvature

The Modal Curvature Method (MCM) is a technique based on the expanded mode shape monitoring theory, which concerns the second derivative of mode shapes. The method was first developed by Pandey et al. [153] based on the relationship between curvature and flexural stiffness (EI). As such, the loss of stiffness due to damage can be sought through monitoring increased modal curvature values. The high level of sensitivity of MCM to damage was demonstrated by [154]. Ho and Ewins [155] improved MCM by amplifying the curvature variations in the Modal Curvature Squared Method (MCSM), which can be employed to more easily discern abnormal changes compared with MCM. However, MCM introduces some drawbacks, such as requiring many sensors to identify higher modes and limited performance due to the number of modes considered in analysis [156]. The central difference approximation used in MCM can magnify the effect of errors in displacement mode shapes. This effect can also amplify high-frequency noise, resulting in an increase in the variance of the extracted damage features [157]. On the other hand, using larger sampling frequency to avoid noise can bring about truncation error [158]. Additionally, calculating the curvatures from measured strain values has shown to be less informative [159]. Given the above drawbacks and to enhance the credentials of MCM, it is usually coupled with other sub-optimal modal parameters, such as natural frequencies [160].

Table 11 presents some of the recent developments in the application of MCM for damage detection in composite structures.

For more information about using MCM for damage detection in composite structures, the readers are referred to [150].

#### 3.2.4. Modal Strain Energy

Modal strain energy is the energy stored in a structure when it undergoes a deformation in its mode shape patterns [156]. Referring to the Euler–Bernoulli beam theory, damage compromises the ability of the structure to store as much energy due to a loss of stiffness, as it would in its healthy state. An assessment of the application of the method to Finite Element (FE) modelled beams demonstrates its superior performance in damage localisation compared with frequency-based damage indicators [164]. According to the same study, modal strains were proposed to be reasonably capable of estimating crack size and, thus, exhibit potential for damage quantification. In another study, Yam et al. [165] indicated the higher sensitivity of strain modes to local structural changes compared with the displacement modes in a tested plate structure. However, the identified strain response of higher modes was not as strong as in lower modes, which limits the use of higher modes strain energy for damage detection. Similar to MCM, the modal strain energy relies on the central difference approximation method that can magnify the effect of noise. Moreover, in order to obtain continuous strain values between sensors, curve fitting techniques must be employed to smooth out the curve resulting in concealed local damage [156].

The application of the modal strain energy method was extended to two-dimensional bending structures by Cornwell et al. [166]. Subsequently, Duffey et al. [167] advanced the method for structures featuring axial and torsional responses. However, both of these methods require numerous sensors and defy from the original relationship between curvature and flexural stiffness. Table 12 presents some of the recent developments of modal strain energy use for the damage detection of composite structures.

For more information about damage detection in composite structures via modal strain energy, the readers are referred to [171].

#### 3.2.5. Modal Damping

Although damping is one structural parameter that can be influenced by damage, it is less commonly considered for damage detection due to its complex nature that does not simply allow for its simulation and study for damage. In a study conducted by Franchetti et al. [172], the nonlinear damping of a concrete structure was identified from ambient vibration responses and further used for damage localisation in the structures without requiring any baseline information available from the undamaged structure. In another study, Mustafa et al. [173] developed an energy-based damping evaluation method for identifying the location of damage in structures. Ay et al. [174] studied the statistical framework of free-vibration of a dynamic system to estimate the damage-induced changes in the overall damping behaviour of the system. Conclusively, damping-based methods are dependent on the specified damping model. For more information about using modal damping for damage detection in composite structures, the readers are referred to [175,176].

#### 3.2.6. Modal Flexibility

Another popular modal parameter for structural damage detection is modal flexibility, which was first proposed by Pandey and Biswas [177] and further applied to bridge structures by Toksoy and Aktan [178]. The modal flexibility method (MFM) is based on the flexibility matrix obtained as the inverse of the structural stiffness matrix. The MFM method can be reconstructed out of fewer modes compared with the stiffness matrix and, thus, has a greater sensitivity to damage, as guaranteed by the reconstruction of the flexibility matrix out of more easily extracted lower modes. Additionally, in light of the higher sensitivity, MFM characterises damage based on a single feature extracted from information embedded in many frequency modes. This has been confirmed in a study conducted by Wang et al. [179], which demonstrated that the advanced damage sensitivity of MFM is superior to other modal-based damage indicators. Moreover, the damage localisation capabilities of MFM were demonstrated in beam and plate structures through a dynamic computer simulation [180]. The good performance of MFM can be attributed to the usage of mass-normalised mode shapes. The displacement pattern of the structure, therefore, can be portrayed per unit applied force by the flexibility matrix. This enhances the damage localisation results, as damage events can be uniformly assessed across different parts of the structure. However, since mass-normalised mode shapes require knowledge about the load effect, MFM’s performance can be compromised by the ambient or unknown conditions effects. Zhang and Aktan [181] employed a hybrid method of MFM and MCM to monitor changes in structural flexibility. The authors devised this method considering that damage increases flexibility and local curvature concurrently at the same location, and therefore, combining these two effects will increase the sensitivity of damage indices. Lu et al. [182] also applied the hybrid MFM–MCM method to a beam and demonstrated the decent sensitivity of the modal flexibility to local damage. However, in the presence of multiple damage, localisation was made difficult, as the flexibility peaks merged together. The results of this study also indicated that, in the case of multiple damage events with varying magnitudes, changes in the flexibility occurred in locations other than the damage sites. Notwithstanding, the results showed that the hybrid MFM–MCM method obtained superior results in localising closely distributed damage and in differentiating between damage events with different magnitudes. Table 13 lists some recent developments of modal flexibility use for damage detection of composite structures.

Additional information about the application of MFM in damage detection of structures can be found in [186].

### 3.3. Frequency Response Function

Unlike modal data, Frequency Response Functions (FRFs) are obtained over a wide range of frequencies, providing more information about damage, and have been widely used as input in optimisation-based model-updating problems [187,188]. Nevertheless, FRFs have also been utilised to obtain damage sensitive features in damage detection problems. For example, in a study conducted by Limongelli [189], a damage sensitive feature based on the difference between the FRF and its spline interpolation was proposed.

The major challenge, however, lies in the choice of a proper frequency range for excitation. Furthermore, the FRF requires knowledge about the excitation force and the corresponding structural response. Transmissibility is a substitute for the FRF, which is defined based on the relationship between two sets of responses and thus is independent of input excitations. Since transmissibility is a local quantity, it is highly sensitive to damage.

Table 14 presents some recent developments of the FRF applications for damage detection in composite structures.

For more information about damage detection using FRFs, the readers are referred to [194,195].

### 3.4. Model Updating

Model updating methods aim to synchronise the responses from a finite element (FE) model of a structure with measured responses by updating the physical parameters of the FE model on an elemental or sub-structural level. Different static and dynamic responses, or a combination of both, have been used in model-updating problems [188,196]. There are generally two types of model-updating methods: (1) sensitivity-based methods and (2) optimisation-based methods.

Table 15 lists some recent advances of model-updating techniques for damage detection of composite structures.

#### 3.4.1. Sensitivity-Based Model Updating Methods

Sensitivity-based model updating methods are set to minimise a penalty function of errors constructed based on the difference between the measured and simulated data [207]. These methods characterise the sensitivity of the FE model parameters by measuring changes in the FE model response caused by a unit change in the model input via iterations. On the other hand, sensitivity-based methods are capable of updating the FE model and of reproducing the measured responses robustly [201]. However, these methods also suffer from modifying the most sensitive element and overlook the element with error. To tackle this problem, it is recommended to localise the errors first and then changes in the corresponding elements to be sought [207].

#### 3.4.2. Optimisation-Based Model Updating Methods

Traditional gradient-based optimisation methods are limited in a sense that they require a good initial value. Modern optimisation-based model updating methods favour the development of computational intelligence techniques, such as the Genetic Algorithm (GA), Artificial Neural Network (ANN), particle Swarm Optimization (PSO), and Artificial Bee Colony (ABC). Since these algorithms do not rely on a fixed mathematical structure for optimisation, they can overcome the aforementioned shortcomings of traditional methods. Moreover, these algorithms are capable of dealing with the uncertainties and insufficient information of structural damage detection problems. The three main categories of population-based metaheuristic algorithms include evolutionary-based, swarm-based, and bio-inspired algorithms [208].

Table 16 indicates some recently developed optimisation-based methods for damage detection of composite structures.

## 4. Advanced Hybrid Vibration Methods

The low-frequency structural vibration-based methods present several advantages, such as (1) the structural responses being relatively easy to interpret, (2) they can be easily applied to complex and larger structures, and (3) they do not necessarily require full access to the structure [11]. Nevertheless, these methods face some limitations. For instance, they have a lower sensitivity to local defects compared with higher frequency-based approaches and require the installation of numerous sensors in order to be able to describe standing wave patterns [218]. Some researchers have employed nonlinear dynamic analysis to feature local defects [219]. Although classical linear methods have been successfully used in various applications [220], they succumb to various properties of nonlinear features, such as high sensitivity to local damage [221] and robustness to environmental effects [222]. Some frequently used nonlinear features for damage identification include the sub-/higher harmonics modulation in the structural response, waveform distortions, correlation between frequency shifts and the excitation amplitude, coherence functions, vibro-acoustic modulation, etc. [222].

### 4.1. Vibro-Acoustic Modulation Techniques

Thanks to the advancement of various NDT methods, the damage detection of composite structures has immensely progressed over the past decades. Some of these methods, which include visual inspection, ultrasonic testing, acoustic emission, X-rays, and vibro-thermography [223], use a web of integrated sensors with the structure under study. Among all methods, guided ultrasonic waves [224] are of particular interest as they require a smaller number of transducers to inspect large structures. Nonlinear damage features have been sought through concurrent application of mechanical vibrations and acoustic waves [225]. A review on such nonlinear interactions can be found in [226].

Vibro-acoustic modulation (VAM) is a nonlinear NDT method that is widely used for structural damage evaluation in different materials, such as composites. The method is based on the application of two types of signals: (1) a more intense low-frequency vibration (pumping signal) and (2) a high-frequency acoustic wave (probing signal). First, the composite component is excited via a low-frequency mechanical signal, and then concurrently, a high-frequency acoustic signal is transmitted through the material. The low-frequency vibration signal causes cyclic opening and closing of microscopic defects, producing modulations in transmitted acoustic signals—a phenomenon termed Contact-Acoustic Nonlinearity [227]. The recorded vibration signal carries information about damage in the form of Higher Harmonics (HH) modulations and Side-Bands (SB). Demodulation techniques are used to isolate the high-frequency content of the recorded signal that has information about damage. VAM is shown to be sensitive to damage severity in complex structures [77].

Numerous studies in the literature have been conducted on the application of VAM in featuring different types of damage in composite materials, such as impact damage [228], delamination [229,230], and debonding [231].

The existing theories of VAM are developed based on one-dimensional spring-mass models [226]. As such, the nonlinear signal of VAM is caused by the nonlinearity of the spring constant, which can stem either from the inherent material nonlinearity or the bilinear behaviour due to the opening and closing of the crack [226]. A generic three-dimensional (3D) body theory of VAM has yet to be developed [232].

### 4.2. Data Analysis Techniques

Traditional signal processing techniques are generally based on the bold assumption that the signals are generated through a stationary and linear process. Table 17 lists some of the advantages and disadvantages of some methods. These methods can result in false information once they are employed for fault detection in signals. The main reason is that the effect of the damage on mechanical responses may be non-stationary, generating a transient effect in the response signals [233]. To deal with non-stationary signals, several advanced time-frequency analysis techniques have been developed and further employed for fault diagnosis of rotating machinery [234]. Time-frequency (TF) methods can provide an improved representation of energy variation in a signal caused by damage and, thus, have attracted much research in the SHM community over the past decades.

The raw data obtained from the deployment of sensors on a structure cannot be used for damage detection on its own and, instead, must be treated to extract meaningful information about the structural health condition. Hence, it is vital to employ some analysis techniques to process the recorded data. One method is to transform the data into various domains whereby hidden information, which is not usually accessible in the raw data, can be extracted. To this end, various frequency-domain analysis (FDA) and time-frequency analysis (TFA) signal processing techniques have been employed. While FDA methods are more suitable for stationary signal analysis, TFA are typically employed to tackle the problem of information extraction out of nonstationary signals. Examples include Short Time Fourier Transformation (STFT), Wavelet Transformation (WT), Empirical Mode Decomposition (EMD), Variational Mode Decomposition (VMD), etc. Some of the most common types of TFA methods employed in composite structures are reviewed in the following sections.

#### 4.2.1. Wavelet Transformation

Wavelet transformation (WT) has been of great interest for SHM due to its high sensitivity to anomalous observations in measured vibration signals. The first studies on the application of wavelet analysis in the damage detection of structures were conducted in the early 1990s during the initial stages of its development. As the first attempt, Surace and Ruotolo [235] employed WT to analyze vibration signals for damage detection. Spatial WT, based on Continuous WT (CWT) with a Haar wavelet, was initially used for crack detection and localisation in beams [236]. Additionally, Sung et al. [237] first employed Discrete WT (DWT) for the damage detection of composite laminates, using Daubechies wavelets for impact damage detection through studying acoustic emission waves. Chang and Chen [238] expanded the work by Wang and Deng [239] on the use of spatial CWT for detection and localisation of damage in Timoshenko beams using Gabor wavelets. The proposed method was further generalised by the authors for spatial damage detection of plate structures [240]. Chang and Chen [241] proposed a CWT-based approach for estimation of crack position and depth in beam-type structures. Rucka and Wilde [242] presented a comparative study on the application of various WT techniques for damage detection of beams and plates through experimental study. To this end, several parameters of WT, including number of the vanishing moments, symmetry and width of the effective support, were considered. The results indicated that Gaussian and reversed bi-orthogonal wavelets were most effective for CWT-based damage identification. Zhong and Oyadiji [243] demonstrated the superiority of Stationary WT (SWT) over Continuous WT (CWT) in terms of computational efficiency by employing symlet wavelets of order 4 for damage detection of simply supported beams, following the same approach taken by [244]. Gökdağ and Kopmaz [245] developed a method based on the calculation of modal assurance criterion through combining CWT and DWT for damage detection of beam-type structures. In all such methods, a metric was sought through sensitivity analysis of wavelet-based methods in damage-identification problems in a bid to estimate the presence and location of damage. Bayissa et al. [246] proposed energetic zeroth-order moment approach based on Daubechies wavelets of order 8 for damage identification of a concrete plate and steel plate girder in a bridge structure. Katunin et al. further developed DWT-based algorithms for damage detection of composite beams [247,248] and plates [249,250] by making use of B-spline wavelets. As such, the application of B-spline wavelets provides higher sensitivity to damage compared with all other compactly supported orthogonal wavelets, such as DWT [249].

Using WT methods in conjunction with other supporting methods has proven to provide better solutions to damage detection problems. For instance, Rucka and Wilde [251] presented a CWT-based algorithm supported by the ANN. Hein and Feklistova [252] used wavelet transform along with ANN for delamination detection in composite beams. Xiang and Liang [253] proposed a two-step 2D DWT-based algorithm along with particle swarm optimisation for damage detection of plate structures. Xu et al. [254] introduced a new damage-detection method using CNN and WT for damage detection of composite structures and verified the results of the proposed method via experimental studies. Sha et al. [255] employed the Teager Energy operator (TEO) in conjunction with WT to process mode shapes of laminated composite beams, termed TEO–WT mode shapes. The results showed that, since each TEO–WT mode shape exhibited a specific sensitivity to damage location, simultaneous detection of multiple damage from a single TEO–WT mode shape is not possible. Wu et al. [256] proposed a novel method for internal delamination detection in carbon fiber-reinforced plastics by combining deep CNN and CWT. The proposed data-driven method can effectively make use of big data without being reliant on complex feature extraction. Su et al. [257] presented a technique for damage localisation and quantification in composites under strong noise background based on synchro-squeezing WT and the stack autoencoder algorithm. Some useful information about feature extraction and selection in dealing with data can be found in [258].

#### 4.2.2. Empirical Mode Decomposition

Empirical Mode Decomposition (EMD) is another time-frequency signal processing technique that can be used to decompose a complex signal into a set of amplitude/frequency modulated and almost orthogonal components, termed intrinsic mode functions (IMFs) [259]. IMFs represent natural oscillation modes that can be deemed as the basis functions extracted from the original signal [260]. Therefore, it is a self-adaptive signal processing algorithm that can be applied to a nonlinear/non-stationary signal to decompose it into its constructive IMFs. It is known that EMD suffers from the mode mixing phenomenon, which can compromise the accuracy of damage-detection methods. Hence, Wu and Huang [261] proposed a new ensemble EMD (EEMD) method to tackle the mode mixing problem of the EMD. Looney et al. [262] introduced a multivariate empirical mode decomposition (MEMD) framework, which is robust to noise and used to produce localised instantaneous frequencies. Leo et al. [263] developed a bi-variate EMD and further applied it for damage detection in composite materials.

Wang et al. [264] proved the equivalence of the computational complexity of EMD and fast Fourier transform (FFT). The researchers further optimised the computational efficiency of EEMD by 1000 times by proposing a fast Hilbert–Huang Transformation (HHT) with an optimized EEMD algorithm. Accordingly, the optimized EEMD method can be considered for real-time impact localisation of composite structures. Other than its mode-mixing problem, EMD also is limited by its ability to only decompose a single measurement data at a time. As such, a multivariate version of the EMD, termed Multivariate EMD (MEMD), was recently proposed, which facilitates the decomposition of multi-channel vibration signals [265,266,267]. Cao et al. [268] developed an ultrasonic signal processing method for non-destructive testing of composite structures by improving the depth evaluation of phased array ultrasonic waves. The developed algorithm is based on a combination of EMD, correlation coefficient analysis, a fuzzy entropy algorithm, and Hilbert transform and, as such, can be regarded as an improved adaptive time-frequency analysis algorithm. Barile et al. [269] used both Wavelet Packet Transform (WPT) and EMD to develop a model for decomposing recorded waveforms. The proposed model reconstructs the decomposed waveforms after excluding the residual signal from the parent waveform and further calculates the energy content of each frequency band of the reconstructed signal. Han et al. [270] extracted damage modes of composite laminates from acoustic emission (AE) signals utilising EEMD and a decorrelation algorithm.

#### 4.2.3. Advancement of

Time–Frequency Signal Analysis and Processing (TFSAP) algorithms

It is generally desirable to have a time–frequency algorithm that enables the decomposition of non-stationary/nonlinear signals contaminated by a high level of noise. This is critical for modal parameter identification from highly noisy vibration data. Variational Mode Decomposition (VMD) is an adaptive signal decomposition algorithm that can be used for the effective decomposition of a non-stationary/nonlinear signal, contaminated by a high level of noise, into a set of mutually independent oscillatory modes (IMFs) [271]. The VMD method has been widely used for fault diagnosis of mechanical systems, and its superiority over other algorithms, such as EMD and EWT, has been proven in several studies [272,273,274]. However, its application in damage detection of composite laminates has yet to be explored.

A recently proposed accurate adaptive signal decomposition method, termed Empirical Fourier decomposition (EFD), can overcome several shortcomings of its preceding algorithms [275]. However, future work needs to be dedicated to exploring the application of this method in damage detection of different structures, such as composite structures.

## 5. Artificial Intelligence

Artificial Intelligence (AI) aims at mimicking human intelligence through developing computer programs for solving complex problems. In early applications, AI was particularly developed to solve rule-based problems. These sorts of problems, which are intellectually difficult for human, were proven to be straightforward for developed AI-based computer programs that are hand-coded by a human expert [276]. Although AI-developed programs are based on human knowledge, they have surpassed human ability in many cases, such as playing chess [277]. Notwithstanding, knowledge-based AI still succumbs to human capabilities in many ‘‘everyday” tasks, such as face recognition, object detection, and speech understanding. Since such tasks are naturally performed by humans based on informal awareness obtained through several experiences about the world, they cannot be explicitly translated to a set of formal rules in a computer program. This is regarded as the most confronting challenge experienced by most AI systems thus far [278], for which the concept of machine learning (ML) was developed to remedy this challenge. An ML algorithm is designed in a way that the program can acquire the required information from data to learn how to fulfill a specific task systematically [279]. To this end, data are required to be pre-processed for extracting and characterising some features in terms of the quality they represent through a procedure termed “feature extraction” [280]. The extracted features are then used to train the ML system to learn how they discriminate different patterns in the data.

### 5.1. Machine Learning

**Figure 6:**
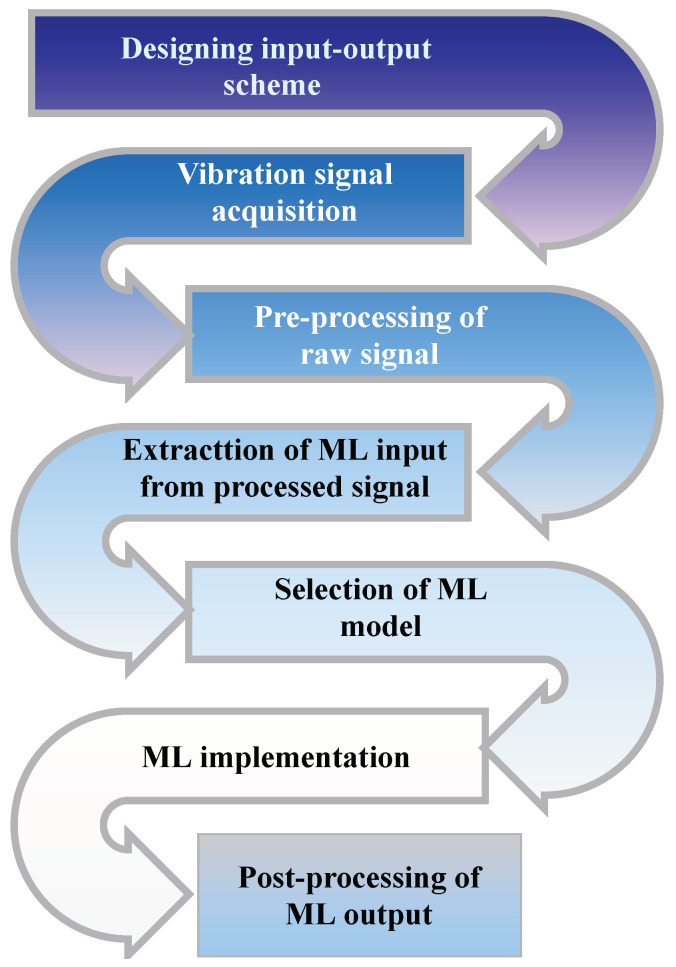
Procedures of training an ML algorithm.

The primarily two classes of ML algorithms include supervised and unsupervised algorithms [281]. Supervised algorithms rely on a human-labeled data for training [282] and aim to establish an optimal mapping of the feature space and the space corresponding to the target values (labels) [283]. Unlike supervised ML algorithms, unsupervised algorithms do not require labeled data, instead their objective is to label data based on the algorithm’s underlying structure [284]. Figure 6 illustrates the procedure of training an ML algorithm. Regression and classification problems are the two types of problems solved by ML algorithms. Some of the recent studies on the application of supervised and unsupervised ML algorithms for different damage detection problems are listed in Table 18.

### 5.2. Deep Learning

As previously discussed, the performance of ML algorithms is mostly reliant on the strength of the extracted features in representing data. It is, however, critical to extract optimal features that can properly characterise properties of the input data in order to simplify the process of establishing the map between the feature and target spaces for ML algorithms [299]. However, it is neither always practical to manually identify the optimal features extracted from the raw data nor very easy to select a proper group of features manually for training [300].

Therefore, Deep learning (DL) methods, such as Deep Neural Networks, have been developed to mitigate the reliance of complex ML applications on hand-crafted features. DL techniques are, thus, a special type of ML algorithm that can extract optimal features directly from raw data without incorporating user intervention. DL systems are hardwired to establish a direct map from raw data to targets without requiring extraction of features *a priori* [301]. Therefore, by learning how to extract high-level and abstract features hierarchically out of simple and low-level learned features [276], DL is able to handle complex problems [302,303,304].

Table 19 lists some reviewed recent studies on the application of DL and ML in SHM of composite structures.

## 6. Smart Structures

**Figure 7:**
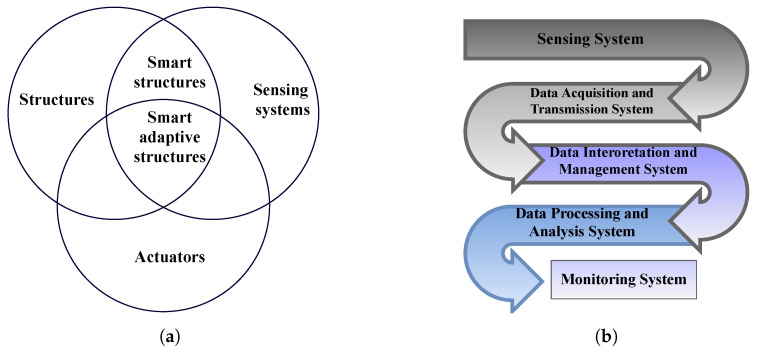
(**a**) Smart structures and smart adaptive structures, and (**b**) implementation of structural health monitoring.

One promising technological advancement of the twentieth century in the realm of SHM is the possibility of integrating sensors and actuation systems with structures (Figure 7a). Similar to the human body, a smart structure is designed to react to external conditions and to change its responses accordingly. The structural system is aimed at performing damage identification and characterisation (recognition, localization, and quantification) as well as to report damage to a control centre for facilitating proper response by the system manager (Figure 7b). To this end, smart structural systems are comprised of several factors, including a host structural material, actuators, a network of sensors, real-time control facilities, and computational appliances. As such, the structure can autonomously monitor the health conditions of the host material in an automatic and continuous fashion, through the following steps:

1. The actuator creates vibration in the structure by inducing strain or displacement.
2. The sensors record the resultant vibration response of the structure.
3. The data recorded by the sensors are transmitted to the control/processor unit.
4. The transmitted data are studied via some computational instrument for damage.

The development of smart structures for damage detection is projected to meet the following goals [313]:

1. Enable the structure to detect damage as soon as it is incurred by the structure;
2. Determine the location and severity of the damage;
3. Predict the remaining service life of the structure; and
4. Alert the operator about the extent to which the performance of the structure was compromised, so that necessary steps can be followed to handle the situation.

Some examples of smart materials include composites with surface-attached or embedded sensors, electrorheological (ER) materials, and magnetorheological (MR) materials [314,315]. Smart structural systems are also common in a range of industries, from aerospace, IT, automobile, and space to the military [316]. As a case in point, one of the most well-known smart system technologies includes composite materials embedded with fiber-optic sensors (FOS) [317], which is utilized in several applications, such as safety-related areas in aircrafts.

### Self-Sensing Composites

The property of a material to sense different factors pertaining to its own conditions, such as stress, strain, damage, and temperature, is termed a self-diagnosing or self-sensing capability. As such, self-sensing composites are capable of sensing their own health condition, which makes this sort of material an excellent choice for conducting continuous SHM of civil engineering structures. Electrical resistivity enables self-sensing composite materials to sense the strain and damage based on the piezoresistivity principle in self-sensing composite materials. To establish piezo-resistivity in composite materials, some conducting elements have to be integrated with the materials. Examples of such conducting elements include short and continuous carbon fibers (CFs), carbon particles, as well as carbon nanomaterials, such as carbon nanofibers (CNFs) and nanotubes (CNTs) [318,319,320]. Moreover, the electrical resistivity of such elements undergoes disruption as soon as the material is subjected to deformation or damage. The results are, however, highly dependent on the amount, type, and distribution of the conducting component. The design flexibility of self-sensing composites is considered one of their main advantages, whereby the type of response can be tailored. Since composites are widely used in civil infrastructures as strengthening materials, integrating self-sensing capability with such materials can strengthen the health monitoring functions of these structures. This further eliminates the required externally deployed sensors on such structures [320].

The following list describes different types of self-sensing composite materials that are used for the SHM of civil infrastructures:

- Polymeric composites [321]
  - Short CF Composites
  - Continuous CF Composites
  - CNT/CNF Composites
- Cementitious composites [322]
  - Short CF Composites
  - Continuous CF Composites
  - CNT/CNF Composites

## 7. Final Remarks

**Figure 8:**
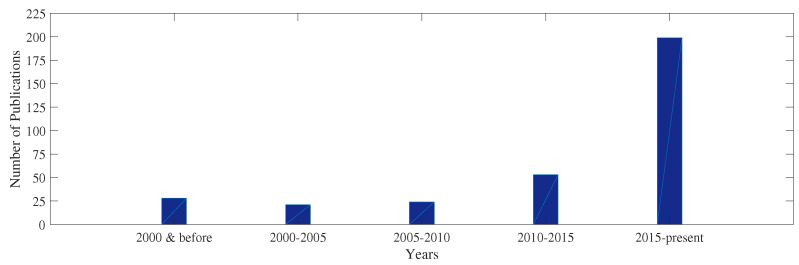
Reviewed number of publications per time period.

In this study, several aspects of composite structures were reviewed, including the types of composite structures, damage mechanisms that can affect such structures, and methods employed for damage detection of composite structures. To this end, 322 papers have been reviewed, with 203 papers were published from 2015 to present, as shown in Figure 8.

Different aspects of the methods for damage detection of composite structures were investigated which include the types of sensing technologies used to this end, the types of recorded data, and various data analysis techniques that can be utilised to interpret the recorded data for extracting information about the health state of the structure under study. Finally, some remarks on the smart structures and self-sensing composites were made. This study, thus, provides a comprehensive reference for any researcher who wants to begin their academic career in the realm of the SHM of composite structures.

## 8. Conclusions and Future Work

This review provides a comprehensive research on the different aspects of SHM of composite structures. First, different types of composite structures were studied, and composite materials were classified based on their compositions. Next, the contribution of each component to different properties of such structures was described. Importantly, this information helps to provide background knowledge about how damage in such structures can progress as these components become defective. Next, different types of damage in such structures were studied and classified based on the component in which they may occur. Since composite materials are highly sensitive to environmental and operational variations (EOV) effects, several environmental effects and their impact on composite materials were fully investigated. Understanding the types of damage and impact of EOV on composite structures can guide an engineer to select a proper damage detection strategy for the SHM of the structure. We demonstrated that different SHM methodologies are effective to unfold a limited range of damage in composites, though some methods, such as AE and NI, are more promising and can reveal a wide range of defects from micro-scale to macro-scale damage. Next, the properties of different sensors employed for the SHM of composite structures were reviewed. As such, it was argued that the proper selection of the sensors depends on the type of data to be recorded for damage detection and is also a function of various other factors that must be considered prior to selecting the type of sensors. Next, different features that can be extracted from vibration signals were reviewed. Such features that are mostly in frequency domains were fully studied along with their advantages and disadvantages. Subsequently, it was demonstrated that advanced damage-detection algorithms developed for composite structures seek nonlinear interactions between a transmitted acoustic signals and mechanical vibration of the structure. As a following argument, these techniques benefit vastly from the development of time-frequency signal processing algorithms. Accordingly, more advanced time frequency features can be extracted for damage detection using these techniques. With the development of ML and DP algorithms, more advanced damage detection methods have bee proposed for composite structures. Therefore, some recent developments made in this area of research were reviewed in this study. Finally, some remarks on the smart structures and self-sensing composites were outlined. Overall, this study provides a comprehensive review on the various aspects of SHM of composite structures and can be referred by any researcher who wants to start research in this exciting area.

## Figures and Tables

**Table 1:** Some recent advancements in SHM of composite structures.

Refs	Method	Description	Model
[14]	Enhanced wavefield imaging	- A new damage index, termed first-to-residual energy ratio (FRER), was developed based on the amplitude signatures and the residual wave components of the first Lamb waves to arrive	A composite plate (CFRP, T300/3231)
[15]	Fiber Bragg Grating (FBG) sensors	- A damage-identification method of CFRP laminated plates based on strain information	CFRP laminated plates
[16]	Edge-reflected Lamb waves	- Structural prognosis is made possible using the proposed method leveraging the multipath reflected Lamb waves	A composite plate (CFRP, T300)
[17]	Frequency domain-based correlation	- The complex frequency domain assurance criterion (CFDAC) was leveraged to develop a domain-based correlation approach	A CFRP laminated plate
[18]	Low-frequency guided waves	- Low excitation frequencies of guided waves (GW) propagation in different types of FE modelling of composite laminates are used for delamination detection - Two new convergence criteria are employed to obtain accurate results	A laminated composite plate
[19]	Correlation function amplitude Vector (CorV)	- The delamination area can be determined through calculation of the relative changes between the CorVs of the intact and damaged composite laminate plates - Combining the method with a statistic evaluation formula resulted in localising damage precisely	A composite sandwich beam
[20]	Continuous wavelet transform and mode shapes	- Higher-order mode shapes or operational deformation shapes (ODSs) were employed for damage detection	A composite plate
[21]	A Lamb wave-based nonlinear method	- An artificial delamination is created in a composite laminate using a thin Teflon sheet to be detected with the proposed Lamb wave-based nonlinear method	A woven fiber composite (WFC) laminate
[22]	Ultrasonic guided waves	- The effective linear and nonlinear guided wave parameters were extracted through Hilbert transform (HT), Fourier transform (FFT), and wavelet transform (CWT) analysis to characterize the delamination length	A composite double cantilever beam (DCBs)

**Table 2:** Some common failure mechanisms along with recommended damage detection methods in composite structures.

Refs	Failure	Description	Method
[39]	Matrix cracking	An NDE method based on propagation of ultrasonic Lamb wave in polymeric composites that is capable of detecting and classifying matrix cracking in the material using artificial intelligence was developed	Method based on guided wave propagation and artificial neural networks
[40]	Fiber cracking	A mixed-mode I/II crack detection criterion was developed for fracture detection of orthotropic materials with arbitrary crack-fiber angle	Augmented Strain Energy Release Rate (ASER)
[41]	Delamination	An image processing methodology, based on digital radiography, was developed to characterize the drilling-induced delamination damage	Image processing

**Table 3:** Influence of environmental conditions on local properties of composite structures. (+) strong, (∘) average, and (−) weak influence. (Dl) Delamination, (T) Temperature, (Dt) Dirt, (M) Moisture, (ER) Electromagnetic Radiation, and (ML) Mechanical Load.

Condition Influence	Notch	Matrix Crack	Fiber Crack	Dl	T	Dt	M	ER	ML
Material Stiffness	∘	∘	+	∘	+	−	+	−	−
Mass	−	−	−	−	−	+	+	−	−
Damping	−	∘	∘	∘	∘	+	∘	−	−
Material Conductivity	+	∘	+	∘	∘	−	∘	∘	∘
Boundary Formation	+	−	−	+	−	∘	−	−	−

**Table 4:** Some references studying the environmental and operational effects.

Effect	Refs	Description
Temperature effects	[56]	Vibration tests conducted on five bridges in the UK indicated that bridge responses are sensitive to the structural temperature
	[57]	The movement of a point in the experimental model with respect to its expected location in the analytical model confirmed a significant expansion of the bridge deck due to the elevated temperature.
	[58]	A 5% variation in the first mode frequency of the bridge, during the 24 h cycle, was detected
	[59]	The frequency–temperature and displacement–temperature correlations using long-term monitoring data were investigated
	[60]	Dempster–Shafer data fusion technique was employed to investigate the correlation between modal data and temperature
	[61]	The regression analysis in conjunction with Principal Component Analysis (PCA) was employed to purify natural frequency from the environmental and operational variations effects
	[62]	The back-propagation neural network (BPNN)-based approach was employed to clean the identified natural frequencies from temperature effects
Boundary condition effects	[63]	The effect of crack and beam lengths on the natural frequencies was investigated
	[64]	The changes in the natural frequencies caused by the freezing bridge supports were investigated
Mass loading effects	[65]	It was noted that heavy traffic on a 46 m long, simply supported plate girder bridge decreased the natural frequencies of the bridge by 5.4%
	[66]	The effect of the traffic mass on the damping ratios becomes evident when the vibration of the deck due to the traffic exceeds a certain level
Wind-induced variation effect	[67]	The alleviated wind velocity can reduce the natural frequency and decrease the modal damping of a suspension bridge
	[68]	A quadratic function can be established to map the vertical amplitude of the bridge response to the wind speed. It was also noted that the damping ratio is dependent on the vibration amplitude

**Table 5:** The advantages, limitations, and ranges of applications of different NDT techniques.

NDTE Technique	Advantages	Limitations	Range of Applications
Neutron imagine (NI) [74]	- Simple - Quick - Economically viable - Easy to handle - Flexible	- Good method for the detection of surface imperfections only - Effective when used to detect macroscopic flaws. Not a good method for micro-damage detection. - Highly subjective and suffers from low repeatability of results and high reproducibility of errors - Requires multiple engineering approaches for subsurface defect detection	- Civil engineering - Aerospace industries - Health monitoring of composite structures
Acoustic emission (AE) [75]	- Good for real-time structural health monitoring - Applies highly sensitive sensors to detect stress waves - Applicable in situSupports large volumes of measurement - Effective for micro-scale damage detection - It is simple, fast, and cost-effective	- Sample must be stressed - Sensitive to surrounding noise - Not effective for thick sample - Hard to explain and characterise damage modes - High-cost in terms of consumables and equipment - Limited in terms of offshore application - High acquisition rates and measurements on test sample are critical - Provides a qualitative damage detection only	- Civil engineering - Automobile industries - Machining - Aerospace industries - Health monitoring of composite structures
Ultrasonic testing (UT) [76]	- Applicable to different material systems - Enables the identification, quantification, and localisation of internal defects - Permits one-sided inspection - Fast scanning - Long-range inspection capability - Suitable for assembly lines - Good for in situ inspection due to portable and compact equipment - Often affordable - Non-ionizing radiation - Minimal preparation requirement - Sensitive to both surface and subsurface discontinuities	- Complex setup and transducer design - Requires skills to interpret multi-modes and complex features - Sensitive to operational and environmental variations - Difficult to identify damage in the close vicinity of probe - Restricted resolution imposed by the limitation of algorithms and computing power - Requires accessible surface to transmit ultrasound	- Material research - Weld inspection - Quality assurance - Bridges - Aerospace industries - Gas trailer tubes - Health monitoring of composite structures
Nonlinear acoustics (NLA) [77]	- A robust method to detect microscopic damage - Capable of fatigue monitoring prior to crack initiation	- Difficult implementation	- Civil engineering - Automobile industries - Medicine - Machining - Aerospace industries - Health monitoring of composite structures
Digital image correlation (DIC) [78]	- Affordable - Easy to implement - Adjustable temporal and spatial resolution - Insensitive to ambient changes	- Requires high-quality speckle patterns - Resolution is limited by speckle pattern - Can be applied for the identification of subsurface defects	- Civil engineering - Automobile industries - Medicine - Machining - Aerospace industries - Health monitoring of composite structures
X-ray radiography and X-ray tomography (XRI) [79]	- Good for different materials - Can identify both surface and bulk damage - Detailed shape of damage can be revealed through 2D and 3D images - Specific resolution at the sub-micron level - High efficiency - Great image-processing ability	- Not good for large structures - Not good for in situ tests - Requires access to both sides of the test specimen - Dangerous ionizing radiation and, therefore, needs protection - Limit access to facilities - Can endanger human health	- Civil engineering - Health monitoring of composite structures
Resistivity [80]	- Self-sensing capability - Real-time monitoring	- Requires electrodes - Can be applied to electrically conductive materials	- Civil engineering - Health monitoring of composite structures
Infrared thermography (IRT) [81]	- Can be implemented real-time - Can visualise damage - Applicable to a wide range of materials - One-sided inspection is possible - Easy and safe operation (non-ionizing radiation) - Fast and cost effective	- Vulnerable and sensitive equipment, not suitable for in situ tests - Restricted by the cost and availability of excitation sources in the field - The accuracy depends on the complexity of the specimen geometries - Data-processing time depends on the computing power and algorithms - Implementation is limited for offshore structure - More automation from footage is needed for crack identification	- Civil engineering - Medicine - Optimising processes - Surveillance - Aerospace industries - Health monitoring of composite structures
Shearography (ST) [82]	- Surface strain measurement via non-contact full-field tests - Flexible to environmental disturbance - Applicable to large composite structures - High-speed capability - Automated inspection capability	- Requires external excitation sources - Sensitive to rigid-body motion - Not ideal for subsurface defect identification - Not resilient to uncertainties	- Civil engineering - Machining - Aerospace industries - Health monitoring of composite structures
Terahertz (THz) [83]	- Robust and repeatable - Great scan rate with imaging - Great accuracy, sensitivity, and resolution - Great penetration depths - Non-ionizing radiation	- Low speed examination - Limited to non-conductive materials - Costly	- Civil engineering - Aerospace industries - Health monitoring of composite structures
Eddy current testing (ET) [84]	- Fast - Contactless	- Can be applied to only electrically conductive materials - Applicable for surface analysis	- Civil engineering - Aerospace industries - Health monitoring of composite structures
Neutron imagine (NI) [85]	- Applicable to different materials - Applicable for in situ tests - Good for both surface and bulk damage detection - Detailed shape of damage can be revealed in 2D and 3D images - High resolution at the sub-millimeter level - High image-processing ability - Provides greater penetration depth than X-rays - High sensitivity to light elements	- Not good for in situ tests - Requires access to both sides - Requires protection against dangerous ionizing radiation - Acquisition efficiency lower than XRI - Access to facilities is limited - More expensive than XRI	- Civil engineering - Automobile industries - Aerospace industries - Health monitoring of composite structures

**Table 6:** Fundamental characteristics of sensors used for damage detection of composite materials.

Specifications	Description
Range	The variation in measurements is limited between a minimum and maximum value, termed the range of a sensor
Sensitivity	The sensors should be sensitive enough to the response of a system to the applied load
Accuracy	The value shown by a sensor might be slightly off by a factor, whereby the accuracy of the sensor can be characterised
Stability	The durability of sensors for long-term condition monitoring of structure
Repeatability	The measurement made by the sensor on the structure subjected to the same load should not vary much from the previous measurements
Energy Harvesting	Energy harvesting capability of sensors is essential for sensors used for long–term condition of structures
Compensation due to change in temperature and other environmental parameters	The signal conditioning feature of the sensors should be capable of reducing the environmental variations effects

**Table 7:** Types of different sensors for damage detection of composite materials.

Measurement	Type	Refs
Displacement	Magnetic optical Ultrasonic Acoustic emission Inductive Capacitive Gyroscope	[107] [108] [109] [110] [111] [112]
Velocity	Magnetic induction Optical Piezoelectric	[113] [114] [115]
Acceleration	Capacitive MEMS Piezoelectric Piezoresistive	[116] [117] [118] [119]
Strain	Piezoresistive Optical	[120] [121]
Force	Piezoresistive Optical	[122] [102]
Temperature	Acoustic Optical Thermoresistive Thermoelectric	[1] [123] [124] [125]
Pressure	Piezoresistive	[126]

**Table 8:** The criteria based on which the type of sensors need to be decided.

Characteristic	Description	Influence
Amplitude range	- Response levels are sensitive to excitations levels	- Sensors can be overloaded or burst by high levels of response - Low levels of response can produce poor data - Certain response levels may not contain damage information - Response level in one frequency range can prevail the response in other ranges
Frequency range	- Excitations in different frequency ranges trigger different response frequencies and deflection patterns in a structural component	- Narrowband data contains short frequency bandwidths - Lower frequency excitations are less capable of revealing small damage - Certain frequencies excitation are more sensitive to damage - Traveling waves combined with vibrations can reveal damage in specific locations
Nature of data	- Constant excitation amplitude produce stationary frequency and phase responses, whereas time-varying excitation amplitude results in nonstationary frequency and phase	- Stationary response data require less data for diagnostics as they are more repeatable - Stationary data also exhibit a cyclic nature that sometimes does not reveal damage in data - Nonstationary response requires averaging as it is not as repeatable - Nonstationary data can expose more types of damage due to its transient nature causing a broader frequency range
Temperature range	- Temperature fluctuation can affect operating components	- Temperature shifts change sensor calibration - Can limit sensors positioning- Sensors and attachment mechanisms can fail due to high/low temperatures
Acoustic excitation	- Air pressure fluctuations can trigger vibration and wave responses	- Acoustic excitations can directly excite sensor housings
Electromagnetic interference	- Converting a measured signal to an electrical signal can produce electric and magnetic fields	- Shielding, such as coaxial cables, is needed to prevent electromagnetic interference - Minimizing the noise effect through preamplification of signals is a common practice

**Table 9:** Characteristics of different modal data employed for damage detection of composite structures.

Features	Types of Damage	Advantages	Disadvantages
Natural frequency	- Delamination - Cracks - Stiffness reduction - Circular holes - Debonding - Impact damage	- Cost effective - Can be conveniently measured from just a few accessible points on the structure - Less sensitive to measurement noise	- Cannot be used alone for damage localisation - Sensitive to environmental and operational variations
Mode shapes and curvature	- Delamination - Cracks - Stiffness reductionCutout - Impact damage	- More sensitive to local damage - Less sensitive to environmental effects	- Require a series of sensors for measurement - They are more prone to measurement noise, compared with the natural frequencies
Modal strain energy	- Delamination - Surface cracks - Stiffness reduction	- Suitable for damage localisation - Effective and practical for detection and quantification of single or multiple damage - Less sensitive to environmental effects	- More sensitive to local damage and small cracks - Not very suitable for damage quantification
Damping	- Delamination - Micro buckling - Debonding - Fiber fracture - Kink bands - Cracks	- Sensitive to even small cracks- Not very sensitive to noise	- Very sensitive to environmental conditions such as temperature
Frequency response function and curvature	- Delamination - Debonding - Impact damage - Cracks	- Suitable for structures with many closely situated eigenvalues - Do not require matching and pairing of the mode shapes - Less sensitive to measurement noise and the accumulation of computation errors	- Measurement of the frequency response function requires a series of sensors

**Table 10:** Some methods developed for damage detection in composite structures using mode shapes.

Ref	Description	Model
[147]	The coefficients of the continuous wavelet transform extracted from the difference between mode shapes of undamaged and damaged structures was used for damage detection. Mathematical techniques were employed to mitigate the edge effect of wavelet transform, to reduce experimental noise in mode shapes, and to identify virtual measuring points. The method was validated by studying steel beams with different cracks sizes and locations experimentally.	Composite beam-type structures.
[148]	Experimentally identified modal parameters were used for damage detection. New damage indicators based on the change in natural frequencies and mode shapes were developed.	A composite cantilever beam
[149]	The mode shape difference curvature (MSDC) analysis method was proposed for estimating damage location and severity in wind turbine blades. The method makes the use of an FEM for dynamic analysis. The mode shape difference curvature (MSDC) information was used for damage detection/diagnosis.	Multi-layer composite material of wind turbine blades
[150]	The proposed method implements online structural health monitoring using modal data used in technologies such as machine learning and artificial intelligence. The commercial FE code Ansys was employed to develop a novel technique, termed node-releasing technique, through FE analysis (FEA) of perpendicular and slant cracks of various depths and lengths in different Unidirectional Laminate (UDL) composite layered configurations.	Laminated composite plates

**Table 11:** Some recent developments in the application of MCM in damage detection of composite structures.

Ref	Description	Model
[161]	The method exploits two-dimensional Chebyshev pseudo-spectral modal curvature to address undesirable properties of the two-dimensional Fourier spectral modal curvature in damage detection. As such, the proposed method is analogous to the two-dimensional Fourier spectral modal curvature. Therefore, it extends the wavenumber domain filtering to the pseudo wavenumber domain.	Composite plates
[162]	A modal frequency curve method combined with wavelet analysis has been proposed for damage detection. It was shown that both numerically and experimentally more robust and unambiguous results can be obtained through using the proposed damage indicator compared with when solely the wavelet coefficients of the studied modes are used. Moreover, the size of defect was identified satisfactorily.	A beam-like structure
[163]	A flexible printed circuit board (FPCB) sensor membrane with polyvinylidene fluoride (PVDF) arrays was developed for accurate extraction of modal curvature to be used for damage detection of in situ aerospace structure. The proposed structure was proven to offer a strong self-sensing performance, where the modal curvature information can be extracted without any calculation of differential equation numerically.	Composite beam structure

**Table 12:** Some recent developments in the application of modal strain energy in damage detection of composite structures.

Ref	Description	Model
[168]	A damage index is proposed based on the ratio of pre- and post-damage modal strain energies. The ratio of modal strain energies of different modes before and after damage was introduced as a damage index. Accordingly, the local areas of the structure was scanned through moving the developed damage indices.	Cylinder
[169]	The mathematical fundamentals of a modal strain energy method was developed and then numerically tested when data were contaminated by 5% noise. The proposed method was proved more accurate, convergent, and efficient when compared with its predecessors.	A beam structure
[170]	A damage detection method based on genetic algorithm and finite element model updating was developed. The proposed objective function was developed based on weighted strain energy. It was shown that the proposed objective function is more sensitive to damage when compared with other methods.	Laminated composite plates

**Table 13:** Some recent developments using modal flexibility in damage detection of composite structures.

Ref	Description	Model
[183]	Two vertical and lateral damage indexes based on the MFM was proposed for damage detection and localisation in the main cables and hangers of a suspension bridge. The proposed vertical damage index requires only the first few modes to accurately detect damage in real suspension bridges.	A suspension bridge
[184]	The MFM was employed to evaluate its performance using the displacement of nodes for damage detection According to the obtained results, the modal flexibility method was capable of damage detection through the displacement of nodes.	A honeycomb composite beam structure
[185]	The MFM was employed for damage detection of cantilever beam-type structures through estimation of the damage-induced inter-storey deflection (DIID). The proposed approach can directly identifies damage location(s) as it relies on a clear theoretical base and does not require an FEM.	Cantilever beam-type structures

**Table 14:** Some recent development in applications of FRFs for damage detection in composite structures.

Ref	Description	Model
[190]	A method based on the modelling of nonlinear Auto-Regressive Moving Average with eXogenous Inputs (NARMAX) and the Nonlinear Output Frequency Response Functions (NOFRFs)-based analyses was proposed for damage detection	Plate structures
[191]	Artificial neural networks were employed to develop a damage detection method using FRFs. The proposed method is capable of nonlinear damage detection effectively when the excitation is set at a specific level	A three-story structure
[192]	A Frequency Response Function (FRF)-based damage detection strategy based on the usage of measured FRF was proposed. Graphical diagrams were used to identify the exact location of defective element(s)	Cantilever beam-type structures
[193]	Three Fractal Dimention (FD)-based damage indices, i.e., Higuchi, Katz, and Sevcik, based on the FD analysis of FRF data in frequency domain were proposed	Beam-type structures
[188]	A modified sensitivity equation was proposed to solve the problem of damage detection in structures with closely situated eigenvalues. The capability of the proposed method in damage detection of structures with closely situated eigenvalues was demonstrated when incomplete noisy measurements were used.	Three-layered laminated composite plate

**Table 15:** Different types of features employed in some recent model-updating techniques for damage detection of composite structures.

Methods	Features	Refs
Conventional model updating	- FRFs - Frequencies and mode shape - Dynamic strain - Accelerations - Static strains and displacements	[197] [198] [199] [200] [201]
Substructuring techniques	- Frequencies and mode shapes - Accelerations	[202] [203]
Regularisation techniques	- Accelerations - Frequencies and mode shapes - Frequencies	[204] [205] [206]

**Table 16:** Different types of features employed in some recent optimisation-based methods for damage detection of composite structures.

Algorithms	Features	Refs
GA	- Mode shapes and stiffness matrix - Natural frequencies - Natural frequencies and accelerations	[209] [210,211] [212]
DE	- Mode shapes - Natural frequencies and mode shape	[213] [214]
PSO	- Natural frequencies and mode shapes - Frequency response function	[215] [215]
ABC	- Natural frequencies and mode shapes - Natural frequencies	[216] [217]

**Table 17:** The advantages and disadvantages of frequency domain versus time domain damage-detection methods.

Methods	Advantages	Disadvantages	Feature
Frequency Domain (FD)	- Simple and rapid identification - Can be coupled with a half power bandwidth approach for damping ratio extraction - They are an accurate, while simple, method for system identification and are widely used in structural modal analysis - Can be used in output-only methods for identifying system parameters - They are appropriate technique for information extraction from closely spaced modes	- Are limited in terms of frequency resolution of the estimated spectral data - They are inaccurate and unreliable for the analysis of nonlinear/non-stationary signals - They can provide resolution in low-frequency ranges, and therefore, fewer numbers of modes can be incorporated - Cannot be used to detect the modal parameters in cable-stayed bridges	- Peak picking (PP) - Complex mode indication function (CMIF) - Least squares complex frequency - domain (LSCF)
Time Domain (TD)	- They are more appropriate for continuous monitoring - Extracted information are more complete compared with FD methods - They can provide resolution in larger frequency ranges, and therefore, a large number of modes can be incorporated - Higher computational complexity than FD methods - They are direct methods and, therefore, are not reliant on any data pre-processing stage to work out correlation functions	- The results can be unreliable for a pair of closely spaced natural frequencies - Generated data from output-only modal analysis can be more scattered - Cannot detect damage for earthquake induced excitation - Require human judgment	- Natural excitation technique (NExT) - Auto-regressive moving average (ARMA) - Subspace system identification (SSI) - Canonical variate analysis (CVA) - Numerical algorithms for state space/subspace system identification (N4SID) - Multivariable output error state-space (MOESP) - Data-driven subspace system identification (SSI-DATA) - Covariance-driven subspace system identification (SSI-COV)

**Table 18:** Some studies on the application of supervised/unsupervised ML algorithms in structural damage-detection problems.

Methods	Advantage	Disadvantage	Input–Output
Supervised learning	- Commonly ML algorithms - Identify Level 1 to 3	- Needs features obtained from both undamaged and damaged states of the structure - The performance depends on the model accuracy	- Frequencies and mode shapes—stiffness reduction [285] - FRF—structural condition monitoring [286] - Dynamic displacement—joint connection damage [287] - Frequencies—damage in a steel-girder bridge model [288] - Acceleration under random excitation—damage in a steel girder-bridge model [289] - Fourier amplitude spectrum of wind-induced acceleration—damage from loosening its connection bolts [290] - Image vectors converted from acceleration—damage detection in hanger cables [291] - Wavelet energy spectrum—multi-pattern anomalies [292] - AR coefficients and residual errors of the statistical parameters—structural condition monitoring [293]
Unsupervised learning	- Needs features of the intact state of a structure - Employed for generating class-information about different modes of failures	- Limited to Level 1 damage identification	- Time-series displacements and rotations—structural condition monitoring [294] - Accelerations from passing vehicle—detecting small stiffness reductions [295] - Frequency domain of ambient vibration—condition monitoring of a railway bridge [296] - Crest factor and T-continues WT extracted—structural condition monitoring [297] - Random acceleration responses—novelty detection [298]

**Table 19:** Some review papers on the application of DL and ML in SHM of composite structures.

Refs	Method	Description	Model
[305]	Deep Learning	- A basalt fiber-reinforced polymer (BFRP) pipeline system was analysed. - Long-gauge distributed fiber Bragg grating (FBG) sensors were used to collect data	Fiber-reinforced polymer (FRP) composite pipeline
[306]	Deep Learning	- A damage-assessment algorithm for composite sandwich structures was developed - The full-field vibration mode shapes and deep learning were employed to this end	Composite sandwich structures
[307]	Deep Learning	- Deep learning was exploited for quantitative assessment of visual detectability of different types of damage in in-service laminated composite structures	Laminated composite structures such as aircraft and wind turbine blades
[308]	Deep Learning	- Labeled damaged data was generated through FE models for a pin-joint composite truss structure - A model-based approach for the data acquisition problem was employed	A pin-joint composite truss structure
[309]	Artificial Neural Network (ANN)	- The fast convergence speed of gradient descent (GD) techniques of ANN and the global search capacity of evolutionary algorithms (EAs) were exploited for network training	Laminated composite structures
[310]	Artificial Neural Network (ANN)	- A new modified damage indicator combined with ANN was proposed - Local Frequency Response Ratio (LFCR) was improved through a transmissibility technique	Laminated composite structures
[311]	Machine learning	- The possibility of damage detection through monitoring acoustic emission (AE) signals generated in minicomposites with elastically similar constituents was demonstrated	Unidirectional SiC/SiC composites
[312]	Deep autoencoder	- Ultrasonic Lamb waves data were used to develop a robust fatigue damage detection method via deep autoencoder (DAE)	Composite structures

## Data Availability

Not applicable.
